# Prescription pattern and effectiveness of antihypertensive drugs in patients with aortic dissection who underwent surgery

**DOI:** 10.3389/fphar.2023.1291900

**Published:** 2023-11-10

**Authors:** Kuang-Ming Liao, Chuan-Wei Shen, Yun-Hui Huang, Chun-Hui Lu, Hsuan-Lin Lai, Chung-Yu Chen

**Affiliations:** ^1^ Chi Mei Medical Center, Chiali, Taiwan; ^2^ School of Pharmacy, Kaohsiung Medical University, Kaohsiung, Taiwan; ^3^ Department of Pharmacy, Taipei Tzu Chi Hospital, Buddhist Tzu Chi Medical Foundation, New Taipei City, Taiwan; ^4^ Institute of Medical Science and Technology, National Sun Yat-Sen University, Kaohsiung, Taiwan; ^5^ Division of Pharmacy, Kaohsiung Armed Forces General Hospital, Kaohsiung, Taiwan; ^6^ Department of Medical Research, Kaohsiung Medical University Hospital, Kaohsiung, Taiwan; ^7^ Department of Pharmacy, Kaohsiung Medical University Hospital, Kaohsiung, Taiwan

**Keywords:** aortic dissection, prescription patterns, antihypertensive drugs, β-blockers, angiotensin-converting enzyme inhibitor, angiotensin receptor blocker, calcium channel blocker

## Abstract

**Background:** Surgical patients with aortic dissection often require multiple antihypertensive drugs to control blood pressure. However, the prescription pattern and effectiveness of antihypertensive drugs for these patients are unclear. We aimed to investigate the prescription pattern and effectiveness of different classes of antihypertensive drugs in surgical patients with aortic dissection.

**Methods:** Newly diagnosed aortic dissection patients who underwent surgery, aged >20 years, from 1 January 2012 to 31 December 2017 were identified. Patients with missing data, in-hospital mortality, aortic aneurysms, or congenital connective tissue disorders, such as Marfan syndrome, were excluded. Prescription patterns of antihypertensive drugs were identified from medical records of outpatient visits within 90 days after discharge. Antihypertensive drugs were classified into four classes: 1) β-blockers, 2) calcium channel blockers (CCBs), 3) renin–angiotensin system, and 4) other antihypertensive drugs. Patients were classified according to the number of classes of antihypertensive drugs as follows: 1) class 0, no exposure to antihypertensive drugs; 2) class 1, antihypertensive drugs of the same class; 3) class 2, antihypertensive drugs of two classes; 4) class 3, antihypertensive drugs of three classes; or 5) class 4, antihypertensive drugs of four classes. The primary composite outcomes included rehospitalization associated with aortic dissection, death due to aortic dissection, and all-cause mortality.

**Results:** Most patients were prescribed two (28.87%) or three classes (28.01%) of antihypertensive drugs. In class 1, β-blockers were most commonly used (8.79%), followed by CCBs (5.95%). In class 2, β-blockers+CCB (10.66%) and CCB+RAS (5.18%) were the most common drug combinations. In class 3, β-blockers + CCB+RAS (14.84%) was the most prescribed combination. Class 0 had a significantly higher hazard of the composite outcome (HR, 2.1; CI, 1.46–3.02; *p* < 0.001) and all-cause mortality (HR, 2.34; CI, 1.56–3.51; *p* < 0.001) than class 1. There were no significant differences in hazards for rehospitalization associated with aortic dissection among classes.

**Conclusion:** Among operated patients with type A aortic dissection, no specific type of antihypertensive drug was associated with a better outcome, whereas among those with type B aortic dissection, the use of β-blockers and CCBs was related to a significantly lower risk of the composite outcome.

## Introduction

Aortic dissection is a life-threatening disease defined as a tear in the intimal layer of the aortic wall, which leads to a separation of the intima and the adventitia through the penetration of the media from the blood (Nienaber et al., 2016). Aortic dissection occurs more frequently in men and older people, with the mean age of occurrence being 65 years (Yeh et al., 2015). In addition, people with hypertension, dyslipidemia, and congenital connective tissue disorders, such as Marfan syndrome, are at a higher risk of aortic dissection (Akutsu, 2019).

Patients diagnosed with aortic dissection receive inpatient management, including surgical treatment and medical treatment. The need for surgical treatment depends on the severity and location of the dissection, the complications of the aortic dissection, and the risk of undergoing the surgery.

One study analyzing data from the International Registry of Acute Aortic Dissection reported that for type A aortic dissection, the in-hospital mortality rates after undergoing surgery and receiving medical treatments were 26% and 58%, respectively, whereas the in-hospital mortality rates were 31.4% and 10.7% for type B aortic dissection, respectively (Booher et al., 2013). Medical treatment is required to control blood pressure and heart rate to decrease the stress on the aortic wall in both operated and non-operated patients. According to guidelines from the American College of Cardiology Foundation (ACCF)/American Heart Association (AHA), European Society of Cardiology (ESC), and Japanese Circulation Society (JCS), β-blockers are recommended as first-line treatments (Hiratzka et al., 2010; JCS Joint Working Group, 2013; Erbel et al., 2014). Patients with aortic dissection are often prescribed more than two antihypertensive drugs to control blood pressure. The efficiency and prescription pattern of patients combining different numbers of classes of antihypertensive drugs are still unclear in patients with aortic dissection who undergo surgery.

There is limited epidemiological and pharmacoepidemiological information on aortic dissection both in Taiwan and worldwide. Most published studies involve aortic dissection patients from a long time ago or have a limited sample size (Mészár et al., 2000; Clouse et al., 2004; Yu et al., 2004; Howa et al., 2013; Pacini et al., 2013; Melvinsdottir et al., 2016; Evangelista et al., 2018). Until now, population-based studies have been limited, and the inclusion period was extended only up to 2012 (Yeh et al., 2015). Moreover, aortic dissection-related outcomes differ between patients who undergo surgery and those who do not (Evangelista et al., 2018). In our previous study, we published data on the prescription patterns and effectiveness of antihypertensive drugs in non-operated aortic dissection patients (Huang et al., 2023). The aim of our current study is to investigate the prescription patterns and effectiveness of antihypertensive drugs in aortic dissection patients who have undergone surgery.

## Materials and methods

### Data sources

The National Health Insurance (NHI) program was launched in 1995, covering approximately 99.6% of the Taiwanese population. The National Health Insurance Research Database (NHIRD) contains various kinds of medical admission records, including outpatient records, inpatient records, and emergency records. Information on disease diagnosis, medication use, images, operations, and other medical procedures is also available in the database. Therefore, the NHIRD is a powerful and representative data source for practicing medical research in Taiwan. This study was conducted with the full population database of the NHIRD from 1 January 2011 to 31 December 2019. Patients with newly diagnosed inpatient aortic dissection between 2012 and 2017 and older than 20 years were included in this study. We deduced the aortic dissection type with the location in which the surgery had been performed according to the NHI surgery codes and stratified aortic dissection patients into two groups in this part, including group A (operated type A aortic dissection) and group B (operated type B aortic dissection). We excluded 1) missing medical records and missing demographic data, including age, sex, premium insurance, and city, as well as patients with uncertain sex; 2) comorbidity of aortic aneurysm and congenital diseases of connective tissue disorders; 3) death before discharge; 4) mortality, rehospitalization associated with aortic dissection, or referral to aortic surgery within 90 days after discharge; and 5) patients without computed tomography (CT), transesophageal echocardiography (TEE), or magnetic resonance imaging (MRI) scans. This retrospective cohort study was used to investigate the characteristics, prescription patterns, and drug utilization of antihypertensive drugs in aortic dissection patients.

### Index date

Index admission was defined as the earliest inpatient diagnosis date of aortic dissection between 1 January 2012 and 31 December 2017. Index discharge was the date of discharge after hospitalization for aortic dissection.

### Comedication

Patients’ inpatient comedications included antihypertensive drugs, and the number of classes of antihypertensive drugs, statins, antidiabetic, antiplatelet, and anticoagulant agents were further analyzed. Antihypertensive drugs were classified into four classes: 1) β-blockers; 2) calcium channel blockers (CCBs); 3) renin–angiotensin system (RAS) agents, including angiotensin-converting enzyme inhibitors (ACEIs), angiotensin II receptor blockers (ARBs), and renin inhibitors; and 4) other antihypertensive drugs.

### Comorbidity

Patients’ comorbidities were identified within 1 year before the index admission (including the index admission) with at least two outpatient diagnoses or one inpatient diagnosis of a disease according to the International Classification of Diseases, Ninth Revision, Clinical Modification (ICD-9-CM) and Tenth Revision (ICD-10-CM) codes.

### Prescription pattern

Prescription patterns of antihypertensive drugs in AD patients were identified by the medical records of outpatient visits within 90 days after discharge. Antihypertensive drugs were first classified into 10 categories: 1) β-blockers, 2) CCBs, 3) ACEIs, 4) ARBs, 5) renin inhibitors, 6) diuretics, 7) vasodilators, 8) α2-agonists, 9) α-blockers, and 10) other drugs.

### NHI code of aortic dissection surgery

The operation situation was detected at index admission and identified with NHI codes, as shown in the following paragraph. According to the surgical site, we can further deduce the aortic dissection type of the operated patients.

### Outcome measurement

We observed the frequency of 10 categories of antihypertensive drugs being prescribed, and 10 categories of antihypertensive drugs were classified into four main classes. The frequency of different combinations of the four classes of antihypertensive drugs was observed. The cohorts were further divided into five classes, including drug non-users (class 0) and drug users prescribed drugs from 1, 2, 3, or 4 classes of antihypertensive drugs (class 1–class 4). The primary outcome was a composite outcome of rehospitalization associated with aortic dissection, referral for aortic surgery, all-cause mortality, and death due to aortic dissection, which is stratified by classes 1–4.

### Statistical analysis

Continuous variables are presented as the means with their corresponding standard deviations. Categorical variables are presented as frequencies and percentages. Univariate and multivariable Cox proportional hazard models were employed. These models are used in the analysis to assess the association between predictor variables and an outcome. Hazard ratios (HRs) and 95% confidence intervals (CIs) were calculated.

The multivariable Cox proportional hazard model was adjusted for covariates. These covariates were selected through stepwise multiple regression analyses. This step helps to control for potential confounding factors. Important risk factors associated with the primary outcome were included in the model. These risk factors included variables such as age, sex, and various comorbidities (e.g., hypertension, hyperlipidemia, diabetes mellitus, heart failure, coronary artery disease, cerebrovascular disease, chronic kidney disease, and chronic obstructive pulmonary disease). A significance level of *p* < 0.05 was used to determine statistical significance. If the *p*-value associated with a particular comparison was less than 0.05, the association was considered statistically significant. In models involving multiple comparisons, the *p*-values were adjusted using the Bonferroni correction. This correction helped reduce the risk of false-positive findings when multiple statistical tests were conducted. The data were processed and analyzed using SAS software version 9.4 (SAS Institute Inc., Cary, NC, United States).

## Results

**FIGURE 1:**
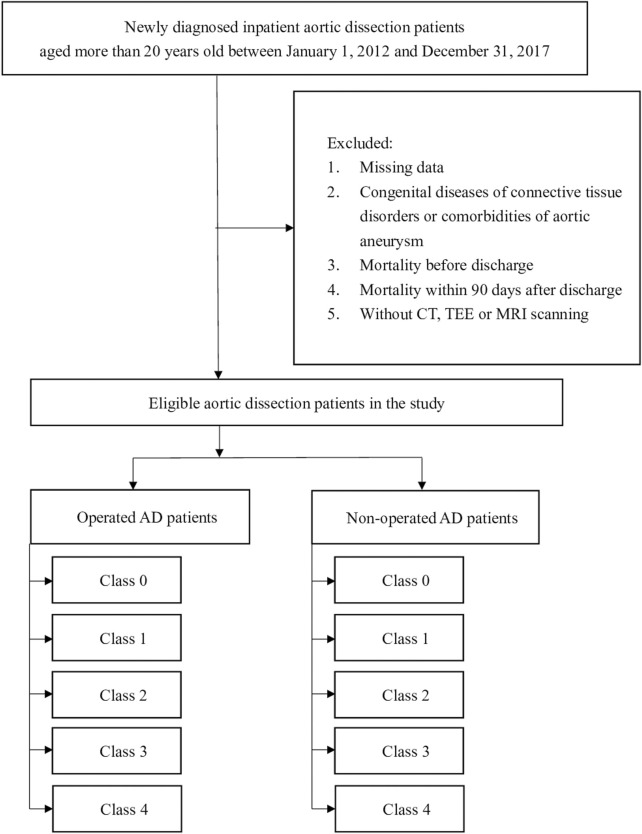
Flowchart of the patient enrollment process of the study cohort.

In total, 11,080 newly diagnosed aortic dissection patients were included in this study. Patients with missing data (*n* = 171), comorbidities of aortic aneurysm and congenital diseases of connective tissue disorders (*n* = 1179), death during hospitalization (*n* = 1394), mortality within 90 days after discharge (*n* = 1219), or without imaging records of CT or MRI (*n* = 192) were excluded from the study (Figure 1). The aortic dissection cohort contained 6,925 patients, and 3,932 patients did not undergo surgery. Among the patients who underwent surgery, there were 1,821 group A patients and 1,172 group B patients. The characteristics of the aortic dissection patients who underwent surgery are presented in Table 1. Regarding inpatient medication, CCB was the most commonly used antihypertensive drug (85.92%), followed by β-blockers (78.04%). The average number of classes of antihypertensive drugs used by inpatients was three classes, and group A used more classes of antihypertensive drugs (3.38 classes). The percentages of prescribed antiplatelet, anticoagulant, antidiabetic agents, and statins were 28.30%, 8.68%, 29.66%, and 12.00%, respectively.

**TABLE 1:** Characteristics of aortic dissection patients, stratified by groups.

Variable	Overall (*n* = 2993)	Group A (*n* = 1821)	Group B (*n* = 1172)
Age, mean, year (SD)	63.54 (14.55)	58.96 (12.38)	59.71 (13.89)
Age-group, N (%)			
<40	176 (5.88)	89 (4.89)	87 (7.42)
40–64	1,778 (59.4)	1,128 (61.94)	650 (55.46)
≧65	1,039 (34.71)	604 (33.17)	435 (37.12)
Sex, N (%)			
Male	2,127 (71.07)	1,220 (67.00)	907 (77.39)
Female	866 (28.93)	601 (33.00)	265 (22.61)
Geographic area, N (%)			
North	1,497 (50.02)	920 (50.52)	577 (49.23)
Middle	579 (19.35)	343 (18.84)	236 (20.14)
South	858 (28.67)	517 (28.39)	341 (29.1)
East	59 (1.97)	41 (2.25)	18 (1.54)
Urbanization, N (%)			
Urban	1,532 (51.19)	942 (51.73)	590 (50.34)
Suburban	1,193 (39.86)	721 (39.59)	472 (40.27)
Rural	268 (8.95)	158 (8.68)	110 (9.39)
Insurance premium, N (%)			
≦22,800 NTDs	1,801 (60.17)	1,094 (60.08)	707 (60.32)
>22,800 NTDs	1,192 (39.83)	727 (39.92)	465 (39.68)
Comedication, N (%)			
β-Blocker	2,543 (84.96)	1,565 (85.94)	978 (83.45)
CCB	2,812 (93.95)	1,712 (94.01)	1,100 (93.86)
RAS^a^	1,998 (66.76)	1,165 (63.98)	833 (71.08)
Others	2,690 (89.88)	1,716 (94.23)	974 (83.11)
Number of classes of antihypertensive drugs, mean (SD)	3.35 (0.91)	3.38 (0.86)	3.31 (0.96)
Antiplatelet	1,093 (36.52)	652 (35.8)	441 (37.63)
Anticoagulant	443 (14.8)	339 (18.62)	104 (8.87)
Antidiabetic agent	1,462 (48.85)	1,013 (55.63)	449 (38.31)
Statin	262 (8.75)	152 (8.35)	110 (9.39)
Comorbidity, N (%)			
Hypertension	2,391 (79.89)	1,431 (78.58)	960 (81.91)
(Continued) variables	Overall (*n* = 2993)	Group A (*n* = 1821)	Group B (*n* = 1172)
Hyperlipidemia	521 (17.41)	316 (17.35)	205 (17.49)
Diabetes mellitus	425 (14.2)	241 (13.23)	184 (15.7)
Heart failure	202 (6.75)	126 (6.92)	76 (6.48)
Atrial fibrillation	175 (5.85)	118 (6.48)	57 (4.86)
Coronary artery disease	479 (16)	286 (15.71)	193 (16.47)
Cerebrovascular disease	565 (18.88)	381 (20.92)	184 (15.7)
Chronic kidney disease	251 (8.39)	133 (7.3)	118 (10.07)
Chronic obstructive pulmonary disease	190 (6.35)	103 (5.66)	87 (7.42)
Asthma	114 (3.81)	62 (3.4)	52 (4.44)
Sleep apnea	13 (0.43)	8 (0.44)	5 (0.43)
Pheochromocytoma	5 (0.17)	1 (0.05)	4 (0.34)
Cystic kidney disease	11 (0.37)	7 (0.38)	4 (0.34)
Obesity	27 (0.5)	8 (0.44)	7 (0.6)
External cause of injury	14 (0.47)	8 (0.44)	6 (0.51)
Charlson comorbidity index score, mean (SD)	1.98 (1.33)	1.95 (1.28)	2.03 (1.41)
Location of AD, N (%)^b^			
UAD	82 (2.74)	53 (2.91)	29 (2.47)
TAD	1,724 (57.6)	1,215 (66.72)	509 (43.43)
AAD	75 (2.51)	8 (0.44)	67 (5.72)
TAAD	1,112 (37.15)	545 (29.93)	567 (48.38)

^a^ RAS: drugs acting on the renin–angiotensin–aldosterone system, including angiotensin-converting enzyme inhibitors (ACEIs), angiotensin II receptor blockers (ARBs), and renin inhibitors.

^b^ Location of aortic dissection according to ICD codes; UAD, unspecified site of the aortic dissection; TAD, thoracic aortic dissection; AAD, abdominal aortic dissection; TAAD, thoracoabdominal aortic dissection; CCB, calcium channel blocker; NTD, New Taiwan dollars; SD, standard deviation.

The most common comorbidities of aortic dissection patients were hypertension (82.82%), coronary artery disease (21.26%), hyperlipidemia (19.96%), cerebrovascular disease (17.83%), and diabetes mellitus (16.27%).

The prescription patterns of aortic dissection patients within 90 days after discharge are shown in Table 2. Antihypertensive drugs were classified into 10 categories to observe the utilization of each type of antihypertensive drug in detail. In group A and group B, the most prescribed antihypertensive drugs were β-blockers (74.85% and 69.28%), CCBs (55.90% and 65.27%), and ARBs (40.14% and 51.19%).

**TABLE 2:** Prescription patterns of aortic dissection patients within 90 days after discharge.

Variable	Overall (*n* = 2993)	Group A (*n* = 1821)	Group B (*n* = 1172)
Categories of antihypertensive drugs, N (%)			
β-Blocker	2,175 (72.67)	1,363 (74.85)	812 (69.28)
CCB	1,783 (59.57)	1,018 (55.9)	765 (65.27)
ACEI	62 (2.07)	35 (1.92)	27 (2.30)
ARB	1,331 (44.47)	731 (40.14)	600 (51.19)
Renin inhibitor	7 (0.23)	3 (0.16)	4 (0.34)
Diuretic	879 (29.37)	577 (31.69)	302 (25.77)
Vasodilator	98 (3.27)	43 (2.36)	55 (4.69)
α_2_-Agonist	13 (0.43)	4 (0.22)	9 (0.77)
α-Blocker	333 (11.13)	176 (9.67)	157 (13.4)
Other drugs	0 (0.0)	0 (0.0)	0 (0.0)
Prescription patterns, N (%), stratified by classes			
Class 0	198 (6.62)	107 (5.88)	91 (7.76)
Class 1	646 (21.58)	430 (23.61)	216 (18.43)
β-Blocker	365 (12.2)	253 (13.89)	112 (9.56)
CCB	143 (4.78)	84 (4.61)	59 (5.03)
RAS	45 (1.5)	32 (1.76)	13 (1.11)
Others	93 (3.11)	61 (3.35)	32 (2.73)
Class 2	964 (32.21)	609 (33.44)	355 (30.29)
β-Blocker + CCB	381 (12.73)	241 (13.23)	140 (11.95)
β-Blocker + RAS	179 (5.98)	114 (6.26)	65 (5.55)
β-Blocker + others	118 (3.94)	48 (2.64)	70 (5.97)
CCB+RAS	166 (5.55)	128 (7.03)	38 (3.24)
CCB+others	78 (2.61)	56 (3.08)	22 (1.88)
RAS+others	42 (1.4)	22 (1.21)	20 (1.71)
Class 3	840 (28.07)	505 (27.73)	335 (28.58)
β-Blocker + CCB+RAS	440 (14.7)	245 (13.45)	195 (16.64)
β-Blocker + CCB+others	177 (5.91)	126 (6.92)	51 (4.35)
β-Blocker + RAS+others	122 (4.08)	86 (4.72)	36 (3.07)
CCB+RAS+others	101 (3.37)	48 (2.64)	53 (4.52)
Class 4	345 (11.53)	170 (9.34)	175 (14.93)
β-Blocker + CCB+RAS+others	345 (11.53)	170 (9.34)	175 (14.93)

Antihypertensive drugs were also classified into four main categories to investigate the different combinations of antihypertensive drugs. Patients were grouped into five classes (classes 0, 1, 2, 3, and 4) according to the number of classes of antihypertensive drugs prescribed; patients not using antihypertensive drugs were defined as class 0. Most patients were prescribed two classes (28.87%) or three classes (28.01%) of antihypertensive drugs. In class 1, β-blockers were the most commonly used drugs (8.79%), followed by CCBs (5.95%). In class 2, β-blockers+CCB (10.66%) and CCB+RAS (5.18%) accounted for most common proportions. In class 3, β-blockers + CCB+RAS (14.84%) was the most prescribed combination.

The baseline characteristics of the operated patients before matching, stratified by classes, are shown in Table 3. Among 2,993 patients, 198 (6.6%) belonged to class 0, 646 (21.6%) belonged to class 1, 964 (32.2%) belonged to class 2, 840 (28.1%) belonged to class 3, and 345 (11.5%) belonged to class 4. Before matching, there were several significant differences in the baseline characteristics of intervention groups (classes 0, 2, 3, and 4) and the comparison group (class 1). Patients in classes 2, 3, and 4 were younger (mean ages: 62.07 for class 1, 62.34 for class 0, 60.53 for class 2, 57.32 for class 3, and 53.31 for class 4) than those in class 1. There were fewer males in class 0 and more males in classes 2, 3, and 4. Patients accounted for a lower proportion of comorbidity of hypertension in class 0 and a higher proportion of having comorbidity of hypertension in classes 2, 3, and 4 (% of hypertension patients: 69.97% in class 1, 59.09 in class 0, 81.22% in class 2, 86.55% in class 3, and 90.43% in class 4).

**TABLE 3:** Baseline characteristics of operated aortic dissection patients before matching, stratified by classes.

Variable	Class 0 (*n* = 198)	Class 1 (*n* = 646)	Class 2 (*n* = 964)	Class 3 (*n* = 840)	Class 4 (*n* = 345)	*p*-value
Age, mean, year (SD)	62.34 (14.90)	62.07 (12.85)	60.53 (12.57)	57.32 (12.36)	53.31 (12.11)	<0.001
Age-group, N (%)						<0.001
<40	15 (7.58)	27 (4.18)	41 (4.25)	59 (7.02)	34 (9.86)	
40–64	87 (43.94)	336 (52.01)	563 (58.4)	543 (64.64)	249 (72.17)	
≧65	96 (48.48)	283 (43.81)	360 (37.34)	238 (28.33)	62 (17.97)	
Sex, N (%)						0.029
Male	133 (67.17)	443 (68.58)	676 (70.12)	609 (72.5)	266 (77.1)	
Female	65 (32.83)	203 (31.42)	288 (29.88)	231 (27.5)	79 (22.9)	
Geographic area, N (%)						0.259
North	90 (45.45)	346 (53.56)	481 (49.9)	413 (49.17)	167 (48.41)	
Middle	44 (22.22)	128 (19.81)	196 (20.33)	152 (18.1)	59 (17.1)	
South	61 (30.81)	159 (24.61)	265 (27.49)	260 (30.95)	113 (32.75)	
East	3 (1.52)	13 (2.01)	22 (2.28)	15 (1.79)	6 (1.74)	
Urbanization, N (%)						0.804
Urban	97 (48.99)	325 (50.31)	490 (50.83)	437 (52.02)	183 (53.04)	
Suburban	88 (44.44)	260 (40.25)	380 (39.42)	330 (39.29)	135 (39.13)	
Rural	13 (6.57)	61 (9.44)	94 (9.75)	73 (8.69)	27 (7.83)	
Insurance premium, N (%)						
≦22,800 NTDs	127 (64.14)	396 (61.3)	580 (60.17)	495 (58.93)	203 (58.84)	0.655
>22,800 NTDs	71 (35.86)	250 (38.7)	384 (39.83)	345 (41.07)	142 (41.16)	
Comedication, N (%)						
β-Blocker	136 (68.69)	497 (76.93)	826 (85.68)	751 (89.4)	333 (96.52)	<0.001
CCB	174 (87.88)	585 (90.56)	910 (94.4)	808 (96.19)	335 (97.1)	<0.001
RAS^a^	83 (41.92)	348 (53.87)	599 (62.14)	652 (77.62)	316 (91.59)	<0.001
Others	165 (83.33)	564 (87.31)	874 (90.66)	759 (90.36)	328 (95.07)	<0.001
Number of antihypertensive drugs, mean (SD)	2.82 (1.3)	3.09 (0.99)	3.33 (0.82)	3.54 (0.78)	3.8 (0.7)	<0.001
Antiplatelet	87 (43.94)	253 (39.16)	329 (34.13)	305 (36.31)	119 (34.49)	0.047
Anticoagulant	30 (15.15)	109 (16.87)	159 (16.49)	107 (12.74)	38 (11.01)	0.025
Antidiabetic	115 (58.08)	324 (50.15)	481 (49.9)	404 (48.1)	138 (40)	<0.001
Statin	6 (3.03)	66 (10.22)	84 (8.71)	69 (8.21)	37 (10.72)	0.019
Comorbidity, N (%)						
(Continued) variables	Class 0 (*n* = 198)	Class 1 (*n* = 646)	Class 2 (*n* = 964)	Class 3 (*n* = 840)	Class 4 (*n* = 345)	*p*-value
Hypertension	117 (59.09)	452 (69.97)	783 (81.22)	727 (86.55)	312 (90.43)	<0.001
Hyperlipidemia	22 (11.11)	108 (16.72)	179 (18.57)	149 (17.74)	63 (18.26)	0.146
Diabetes mellitus	34 (17.17)	92 (14.24)	137 (14.21)	111 (13.21)	51 (14.78)	0.699
Heart failure	17 (8.59)	52 (8.05)	52 (5.39)	55 (6.55)	26 (7.54)	0.199
Atrial fibrillation	8 (4.04)	39 (6.04)	72 (7.47)	41 (4.88)	15 (4.35)	0.070
Coronary artery disease	29 (14.65)	126 (19.5)	138 (14.32)	148 (17.62)	38 (11.01)	0.003
Cerebrovascular disease	78 (39.39)	132 (20.43)	175 (18.15)	134 (15.95)	46 (13.33)	<0.001
Chronic kidney disease	21 (10.61)	52 (8.05)	83 (8.61)	64 (7.62)	31 (8.99)	0.693
Chronic obstructive pulmonary disease	19 (9.6)	44 (6.81)	58 (6.02)	56 (6.67)	13 (3.77)	0.094
Charlson comorbidity index score, mean (SD)	2.43 (1.51)	2.09 (1.41)	1.98 (1.3)	1.88 (1.3)	1.77 (1.17)	<0.001

^a^ RAS: drugs acting on the renin–angiotensin–aldosterone system, including angiotensin-converting enzyme inhibitors (ACEIs), angiotensin II receptor blockers (ARBs), and renin inhibitors; CCB, calcium channel blocker; NTD, New Taiwan dollars; SD, standard deviation.

The primary and secondary outcomes of the operated aortic dissection patients after matching are shown in Table 4. Class 0 had a significantly higher hazard of the composite outcome (HR, 2.1; CI, 1.46–3.02; *p* < 0.001) and all-cause mortality (HR, 2.34; CI, 1.56–3.51; *p* < 0.001) than class 1. There were no significantly different hazards between classes for rehospitalization associated with aortic dissection. Compared to class 1, classes 2, 3, and 4 had no significantly different hazards among them for the composite outcome, rehospitalization associated with aortic dissection, or all-cause mortality.

**TABLE 4:** Primary and secondary outcomes of operated aortic dissection patients after matching.

	N	Event	PY	Rate (%)^a^	HR/sub-distribution HR (95% CI)	*p*-value
Composite outcome (primary outcome)^b^						
Class 0	180	82	582	14.09	2.1 (1.46–3.02)	<0.001*
Class 1	180	45	683	6.59	1 (reference)	
Class 2	574	154	2,261	6.81	1.07 (0.85–1.34)	0.586
Class 1	574	146	2,279	6.41	1 (reference)	
Class 3	475	92	1,949	4.72	0.77 (0.58–1.01)	0.058
Class 1	475	116	1,882	6.16	1 (reference)	
Class 4	218	37	845	4.38	1.04 (0.66–1.64)	0.869
Class 1	218	38	905	4.20	1 (reference)	
Rehospitalization associated with aortic dissection^c^						
Class 0	180	14	582	2.41	0.91 (0.44–1.88)	0.797
Class 1	180	15	683	2.20	1 (reference)	
Class 2	574	60	2,261	2.65	1.18 (0.81–1.71)	0.396
Class 1	574	51	2,279	2.24	1 (reference)	
Class 3	475	38	1,949	1.95	0.82 (0.53–1.26)	0.359
Class 1	475	46	1,882	2.44	1 (reference)	
Class 4	218	20	845	2.37	1.08 (0.58–2.03)	0.806
Class 1	218	19	905	2.10	1 (reference)	
All-cause mortality						
Class 0	180	71	625	11.35	2.34 (1.56–3.51)	<0.001*
Class 1	180	35	729	4.80	1 (reference)	
Class 2	574	106	2,416	4.39	1.04 (0.79–1.37)	0.778
Class 1	574	102	2,421	4.21	1 (reference)	
Class 3	475	62	2,037	3.04	0.80 (0.57–1.11)	0.178
Class 1	475	77	2,009	3.83	1 (reference)	
Class 4	218	17	899	1.89	0.89 (0.47–1.68)	0.712
Class 1	218	21	957	2.19	1 (reference)	
Death due to aortic dissection						
Class 0	180	22	761	2.89	1.74 (0.88–3.46)	0.113
Class 1	180	13	786	1.65	1 (reference)	
Class 2	574	32	2,626	1.22	1.19 (0.71–1.98)	0.516
Class 1	574	27	2,635	1.02	1 (reference)	
Class 3	475	21	2,144	0.98	0.96 (0.53–1.75)	0.905
Class 1	475	22	2,157	1.02	1 (reference)	
Class 4	218	5	921	0.54	0.66 (0.21–2.01)	0.462
Class 1	218	8	993	0.81	1 (reference)	

^a^ Rate was calculated as events divided by person-years, presented as %.

^b^ Composite outcome including rehospitalization associated with aortic dissection, referred to aortic surgery, and all-cause death.

^c^ Estimated sub-distribution hazard ratios with the Fine and Gray model. *p < 0.05. HR, hazard ratio; PY, person-year.

In addition to conducting studies among patient groups after matching, the current study performed studies with unmatched patients as sensitivity analyses. Univariate and multivariate Cox proportional hazard models were used to compare classes 0, 2, 3, and 4 with class 1. Considering the multiple comparisons within the model, the Bonferroni correction was used, and a two-sided *p* < 0.0125 was defined as statistically significant. The sensitivity result of the association between the number of classes of antihypertensive drugs in operated patients is shown in Table 5.

**TABLE 5:** Primary and secondary outcomes of operated aortic dissection patients before matching.

	N	Event	PY	Rate (%)^a^	Crude HR (95% CI)	*p*-value	Adjusted/sub-distribution HR^b^ (95% CI)	*p*-value
Composite outcome (primary outcome)^c^								
Class 0	198	91	641.8	14.2	2.24 (1.73–2.90)	<0.001*	2.18 (1.68–2.84)	<0.001*
Class 1	646	164	2,581	6.35	1 (reference)		1 (reference)	
Class 2	964	249	3,817	6.52	1.03 (0.85–1.25)	0.773	1.08 (0.89–1.32)	0.426
Class 3	840	166	3,438	4.83	0.76 (0.61–0.94)	0.013	0.84 (0.68–1.05)	0.131
Class 4	345	55	1,341	4.10	0.65 (0.48–0.88)	0.006*	0.80 (0.58–1.09)	0.151
Rehospitalization associated with aortic dissection								
Class 0	198	14	641.8	2.18	0.98 (0.54–1.76)	0.940	0.99 (0.55–1.79)	0.969
Class 1	646	56	2,581	2.17	1 (reference)		1 (reference)	
Class 2	964	105	3,817	2.75	1.26 (0.91–1.75)	0.157	1.17 (0.84–1.62)	0.362
Class 3	840	86	3,438	2.50	1.15 (0.82–1.61)	0.411	0.99 (0.70–1.40)	0.970
Class 4	345	32	1,341	2.39	1.09 (0.71–1.68)	0.700	0.83 (0.53–1.31)	0.426
All-cause mortality								
Class 0	198	80	685.4	11.7	2.85 (2.14–3.79)	<0.001*	2.57 (1.91–3.45)	<0.001*
Class 1	646	115	2,745	4.19	1 (reference)		1 (reference)	
Class 2	964	159	4,088	3.89	0.93 (0.73–1.18)	0.554	1.04 (0.81–1.33)	0.759
Class 3	840	96	3,642	2.64	0.63 (0.48–0.82)	<0.001*	0.81 (0.61–1.07)	0.138
Class 4	345	23	1,424	1.62	0.39 (0.25–0.61)	<0.001*	0.62 (0.39–0.99)	0.043
Death due to aortic dissection								
Class 0	198	25	835.5	2.99	3.11 (1.83–5.29)	<0.001*	3.02 (1.76–5.21)	<0.001*
Class 1	646	30	2,982	1.01	1 (reference)		1 (reference)	
Class 2	964	42	4,417	0.95	0.95 (0.60–1.52)	0.831	0.92 (0.57–1.47)	0.725
Class 3	840	34	3,805	0.89	0.90 (0.55–1.46)	0.657	0.95 (0.57–1.57)	0.832
Class 4	345	6	1,459	0.41	0.43 (0.18–1.03)	0.059	0.48 (0.20–1.17)	0.105
Rehospitalization associated with aortic dissection^d^								
Class 0	198	14	641.8	2.18	0.98 (0.54–1.76)	0.940	0.82 (0.45–1.5)	0.524
Class 1	646	56	2,581	2.17	1 (reference)		1 (reference)	
Class 2	964	105	3,817	2.75	1.26 (0.91–1.75)	0.157	1.16 (0.84–1.62)	0.373
Class 3	840	86	3,438	2.50	1.15 (0.82–1.61)	0.411	1.01 (0.71–1.42)	0.978
Class 4	345	32	1,341	2.39	1.09 (0.71–1.68)	0.700	0.84 (0.54–1.31)	0.450

^a^ Rate was calculated as events divided by person-years, presented as %.

^b^ Adjustment for covariates selected by stepwise multiple regression analyses and important risk factors associated with aortic dissection, including age, sex, and comorbidities including hypertension, hyperlipidemia, diabetes mellitus, heart failure, coronary artery disease, cerebrovascular disease, chronic kidney disease, and chronic obstructive pulmonary disease.

^c^ Composite outcome including rehospitalization associated with aortic dissection, referred to aortic surgery, and all-cause death.

^d^ Estimated adjusted sub-distribution hazard ratios with the Fine and Gray model. *p < 0.0125 (adjusted multiple comparisons with Bonferroni correction). HR, hazard ratio; PY, person-year.

For the composite outcome, class 0 and class 4 had significantly higher (HR, 2.24; CI, 1.73–2.90; *p* < 0.001) and lower crude hazard ratios (HR, 0.65; CI, 0.48–0.88; *p* = 0.006) than class 1. However, after adjustments were made for covariates selected by stepwise multiple regression analyses and important risk factors associated with aortic dissection, only class 0 had a significantly higher risk (adjusted/sub-distribution HR, 2.18; CI, 1.68–2.84; *p* < 0.001) in terms of the composite outcome.

Regarding rehospitalization associated with aortic dissection, compared to class 1, there was no significant difference in the adjusted HRs of any class.

Regarding all-cause mortality and death due to aortic dissection, only class 0 had significantly higher adjusted hazard ratios, with adjusted/sub-distribution HRs of 2.57 (95% CI, 1.91–3.45; *p* < 0.001) and adjusted/sub-distribution HRs of 3.02 (95% CI, 1.76–5.21; *p* < 0.001), respectively, than class 1.

## Discussion

This study was a population-based retrospective cohort study on aortic dissection in Taiwan, with a study period between 2011 and 2019. A total of 11,080 newly diagnosed inpatient aortic dissection patients were included in the study between 2012 and 2017, and 2,993 patients with aortic dissection underwent surgery for further research.

The crude incidence rate was 7.8 per 100,000 patients with aortic dissection in Taiwan in our study. Our incidence rate of aortic dissection was higher than that obtained from the study conducted by Yeh et al. (2015), which included aortic dissection patients between 2005 and 2012 with the NHIRD in Taiwan and reported an incidence rate of 5.6 per 100,000 people. The higher incidence rate in our study may be due to differences in the enrollment criteria for the inclusion of aortic dissection patients between these two studies.

Regarding the characteristics of age and sex, compared to two population-based epidemiology studies in Taiwan, our patients had a similar mean age (63.5 ± 14.6) and ratio of males to females (2.5:1) (Yu et al., 2004; Yeh et al., 2015). However, the age of aortic dissection seemed to decrease over time in Taiwan and may be associated with the increased prevalence of hypertension in young and middle-aged populations from 2005 to 2018 (Pang et al., 2019).

The proportion of patients in group B was significantly higher in our study, as well as in the study by Yeh et al. (2015), than that inthe study conducted by Pacini et al. (2013). This difference may be attributable to the trend in open thoracic surgery shifting toward thoracic endovascular repair (TEVAR) for type B aortic dissection. TEVAR is associated with fewer perioperative aortic events and complications, and lower mortality rates than open thoracic surgery (Cheng et al., 2010; Conrad et al., 2010; Chou et al., 2015; Pang et al., 2019). In addition, the mean age in our study was lower, and the male-to-female ratio was higher, which could be due to the higher prevalence of risk factors associated with aortic dissection, such as hypertension, cardiovascular diseases, or smoking, in the male population in Taiwan (Pan et al., 2001; Wen et al., 2005; Chen et al., 2021).

In comparison to the study conducted by Yeh et al. (2015), the mean age and sex distribution in group A and group B were similar. However, our study had a higher number of patients with comorbidities such as hypertension, hyperlipidemia, or diabetes mellitus. This discrepancy can be attributed to the different identification phases for comorbidities between these two studies. The study conducted by Yeh identified comorbidities in aortic dissection patients 1 year before the index date, whereas our study identified comorbidities both 1 year before and on the index date itself. Another study conducted a population-based retrospective cohort study that included aortic dissection patients from 2001 to 2013 in Taiwan using NHIRD data. This study reported the proportions of comorbidities such as hypertension (81%) and diabetes mellitus (15%) (Chen et al., 2021). In our study, the proportions of these comorbidities were similar (hypertension, 83% and diabetes mellitus, 16%) to their findings.

Overall, studies conducted in Taiwan reported a higher usage of ARB and CCB than that in the Suzuki study. This result may be associated with the changing patterns of antihypertensive drug utilization over time in Taiwan. A pattern analysis study conducted in Taiwan indicated a significant increase in the use of ARB and CCB between 2001 and 2006 (Huang et al., 2013). In addition, according to a review study published in 2019, CCBs were the most prescribed antihypertensive drugs in the management of hypertension in Taiwan, followed by ARBs (Cheng et al., 2020).

Operated aortic dissection patients were mostly prescribed β-blockers, whereas in non-operated aortic dissection patients in our previous study, CCBs were the most prescribed antihypertensive drugs. For the operated aortic dissection patients, their medical treatments were closer to the suggestion from the current guideline, which took β-blockers as the first-line choice (Bossone et al., 2018).

To evaluate the association between the number of classes of antihypertensive drugs and the long-term outcome, we set a 90-day landmark after index discharge to identify prescription patterns of aortic dissection patients during their outpatient visits and excluded patients with any outcome of interest within the 90-day period.

The main outcomes (outcomes conducted after matching classes 0, 2, 3, and 4 with class 1) and secondary outcomes were consistent with the sensitivity analyses (outcomes analyzing the original cohorts without matching) in all aortic dissection patients who underwent surgery.

In our study, as the class number increased from 1 to 4, the age of the patients decreased, and the proportion of males increased. Younger patients with aortic dissection had either uncontrolled hypertension or hypertension with a more severe condition, thus requiring more classes of antihypertensive drugs to achieve optimal blood pressure control. Compared to those in class 1, the risks of the composite outcome and secondary outcomes in operated aortic dissection patients in classes 2, 3, and 4 were not significantly different. It is possible that patients with varying degrees of hypertension received appropriate prescriptions for blood pressure control and had similar long-term outcome risks during follow-up in Taiwan. Operated aortic dissection patients in class 0 had a significantly higher risk of the composite outcome (HR, 2.10; CI, 1.46–3.02) than those in class 1. To further investigate the possible reasons, we conducted *post hoc* analyses to determine if there were differences in outpatient visit adherence within 90 days after discharge between groups and how this factor affected the outcomes. As shown in Supplementary Appendix S1, operated aortic dissection patients in class 0 had significantly fewer outpatient visits than those in class 1. This factor was identified as a protective factor in operated patients in the multivariable Cox proportional hazard model. After adjustments were made for this factor, the HRs decreased from 2.10 to 1.68. These results indicate that outpatient visits may still be important for patients who do not use medications after receiving inpatient surgical treatment.

There were two previous studies that investigated the risks of outcomes between patients using different numbers of classes of antihypertensive drugs, including studies conducted by Sakakura et al. (2009) and Liao et al. (2016). The Liao study included patients with non-operated type B aortic dissection, and the Sakakura study included patients with type B aortic dissection. The study conducted by Liao et al. (2016) was a single-center retrospective cohort study, including 106 non-operated type B aortic dissection patients with an observation period from 2008 to 2013 in Taiwan. The primary endpoint of the study was a composite outcome of all-cause mortality and hospital admission related to aortic dissection. The results showed that compared to class 1, there was no significant difference in the composite outcome in classes 0, 2, 3, or 4. In the comparison of the composite results of our study to those of the study conducted by Liao et al. (2016), the results were similar between all intervention groups and comparison groups. However, there were several differences between our study and the study conducted by Liao et al. (2016). First, the current study was conducted with a national database. Therefore, our study had a much larger sample size and a more comprehensive hospital level. Second, the definitions of composite outcome were different. In our study, the composite outcome included all-cause mortality, rehospitalization associated with aortic dissection, and death due to aortic dissection. In the study conducted by Liao et al. (2016), the composite outcome contained the event of all-cause mortality and hospital admission related to aortic dissection. However, the results of the two composite outcomes may be similar. Third, the definitions of exposure were different. In our study, patients were grouped into classes according to their prescriptions at outpatient visits within 90 days after discharge. In the study conducted by Liao et al. (2016), patients were grouped by the drugs used with MPR≥80 in the whole follow-up period. This may have led to differences in the grouping characteristics of the two studies. Nevertheless, the results of our study and those of the study conducted by (Liao et al., 2016) were similar, which may indicate that the grouping at different time points had similar results. Last, the patient inclusion criteria were different. In our study, with a setting of a 90-day landmark, patients with events occurring within 90 days after discharge were excluded. However, the study by Liao et al. (2016) excluded only patients who died during hospitalization. In our study, the condition of events occurring within 90 days after discharge could not be met. This is the reason that the CIs of the HRs were wide in the study by Liao et al. (2016).

The study conducted by Sakakura et al. (2009) was a single-center retrospective cohort study. A total of 202 type B aortic dissection patients were included with an observation time from 1991 to 2006 in Japan. The endpoint of the study was all-cause mortality. Thus, in the following section, we compare the results of all-cause death in our study to those from the Sakakura study. The Sakakura study showed that class 0 had a significantly higher risk of all-cause mortality (adjusted HR, 9.51; *p* = 0.007) than class 1. Compared to that of patients in class 1, the all-cause mortality of patients using 2, 3, or 4 or more classes of antihypertensive drugs was not significantly different. However, in our study, compared to that in class 1, there was no significant difference in the risk of all-cause mortality in classes 0, 2, 3, or 4. When the results of our study were compared to those of the Sakakura study, the results between the comparison of class 0 and class 1 were different. There are some differences between the two studies. In our study, patients with events occurring within 90 days after discharge were excluded, and patients were grouped according to the prescription patterns of outpatient visits that occurred within this 90-day period. However, in the Sakakura study, patients were grouped based on the prescription of antihypertensive drugs at discharge. Patients were observed from discharge to the death event in the Sakura study and from 90 days after discharge to the death event in our study. This may be the reason for the different results in the comparison of all-cause mortality between class 0 and class 1.

Overall, the results of drug users were similar between our study and the Sakakura study. It was mentioned in the Sakakura study that the blood pressure at discharge was not significantly different between groups. In our study, although blood pressure data were lacking, on the basis of observing similar results, it was possible that there was no significant difference in blood pressure among drug user patients in groups at 90 days after discharge, thus causing the risk of mortality to not be significantly different between intervention groups and comparison groups.

Regarding the effectiveness of different prescription patterns among patients using the same number of classes of antihypertensive drugs, among the operated aortic dissection patients within classes 1, 2, and 3, compared to the control group, no specific combinations led to a significantly better benefit regarding the composite outcome in the multivariate proportional hazard model. Previous observational studies have investigated the effectiveness of β-blockers, ACEIs/ARBs, and CCBs at the same time (Golledge and Eagle, 2008; Suzuki et al., 2012). The study conducted by Suzuki et al. (2012) showed that β-blockers were associated with improved survival, especially in operated type A aortic dissection.

With regard to the results of operated aortic dissection patients in our study, there was no benefit of combination treatment in terms of the composite outcome.

The results of the subgroup analysis are shown in Supplementary Appendix S2, Supplementary Appendix S3. In operated type A aortic dissection patients, within classes 1, 2, and 3, compared to the control group, no specific combinations led to a significantly better benefit. However, among the operated type B aortic dissection patients, the risk of the composite outcome was significantly lower with the use of β-blockers (HR, 0.40; CI, 0.20–0.80; *p* = 0.010) and CCBs (HR, 0.35; CI, 0.17–0.72; *p* = 0.005) within class 1. In addition, although significance was not achieved after Bonferroni correction, the use of drugs acting on the RAS was associated with a lower hazard ratio (HR, 0.19; CI, 0.04–0.92; *p* = 0.039) than the use of the control treatment. Furthermore, within classes 2 and 3, no specific combination was associated with better outcomes than those in the control group.

There are some key points that must be emphasized in the pharmacological treatment for all these cases. The recommendations for the initial management of acute type A aortic dissections are highly consistent across society guidelines. They advocate for therapeutic measures aimed at reducing wall stress to limit the extension of the dissection, thus lowering the risk of developing end-organ damage and rupture. Beta-blockers are recommended as first-line agents, with non-dihydropyridine calcium channel-blocking agents considered as second-line options to control the tension and shear stress over the aortic wall while also aiming for a heart rate of 60 beats per minute or less. In addition, ACEIs and vasodilators may be added to achieve a systolic blood pressure of less than 120 mmHg. The recommendations for acute type B aortic dissections adhere to the same principles, with the primary goal being the reduction of aortic wall stress. It is crucial to manage aortic stress and lower the heart rate effectively.

The appropriate treatment for patients with type A aortic intramural hematoma remains a subject of ongoing debate. Most studies conducted in Western countries have reported a more favorable prognosis for surgical groups than medical treatment (Evangelista et al., 2005; von Kodolitsch et al., 2003). However, several centers, particularly in Japan and Korea, have reported positive outcomes with initial medical therapy, reserving surgical treatment for complicated cases, resulting in an in-hospital mortality rate of less than 10% (Song et al., 2003; Song et al., 2009). These differences in prognosis can be attributed to several significant distinctions between Asian and Western patient cohorts: (I) the majority of studies included a limited number of patients and (II) the reported prevalence of intramural hematoma in the International Registry of Acute Aortic Dissection registry and other Western studies ranged from 5% to 18% (Evangelista et al., 2005; von Kodolitsch et al., 2003), whereas Japanese and Korean series reported a prevalence of over 30% among acute aortic syndrome patients (Song et al., 2003; Song et al., 2009; Choi et al., 2014).

### Strengths

Aortic dissection is a severe disease with low incidence, and previous studies of aortic dissection were limited by their small sample sizes. In the current study, we investigated the characteristics, prescription patterns, and effectiveness of antihypertensive drugs in patients with aortic dissection who underwent surgery by using the NHIRD, which provides updated information on patients with aortic dissection in Taiwan, with a large sample size, and includes multiple hospital levels. Moreover, antihypertensive drugs available in Taiwan were all identified in this study, giving a complete picture of the prescription pattern and drug utilization in Taiwan. Most published studies evaluated the effectiveness of drugs based on the prescription at discharge. In our study, considering the adjustment of the drug use after discharge, we identified the prescription pattern 90 days after discharge.

### Limitations

Restricted to the nature of the NHIRD, detailed information on image examinations was not available to classify aortic dissection patients into type A or type B. Alternatively, we adapted the system addressed from the Pacini study to stratify aortic dissection patients who received surgery into group A and group B.

In this study, we evaluated the overall effectiveness of antihypertensive drugs at the class level. We did not investigate the effectiveness of individual drugs or their dosages. Therefore, the current study was limited to the class effects and the qualitative effects. In addition, smoking was a risk factor associated with aortic dissection, but smoking data were not available from the NHIRD. Thus, we collected and matched/adjusted the comorbidity of chronic obstructive pulmonary disease as a surrogate method. The effectiveness research in our study was limited to the class effects and the qualitative effects. The effectiveness of individual drugs according to dosage may be considered for future research.

We delineate the prescription patterns observed in this population of aortic dissection patients. However, it is important to note a limitation: although we have not obtained an effectiveness profile, patients who did not receive medications during the follow-up period experienced more complications.

## Conclusion

Our current study provides an update on the information on characteristics and prescription patterns and an investigation of the effectiveness of antihypertensive drugs in aortic dissection patients who undergo surgery in Taiwan. The most prescribed classes of antihypertensive drugs are β-blockers, CCBs, and ARBs. The most prescribed drugs within these three classes are bisoprolol, amlodipine, and valsartan. For operated aortic dissection patients not using drugs, adherence to outpatient visitation after discharge may still be important, even if the patient has already undergone surgery to repair the aortic dissection. For operated type A aortic dissection patients, no specific type of antihypertensive drug is associated with a better outcome, whereas for operated type B aortic dissection patients, the use of β-blockers and CCBs is related to a significantly lower risk of the composite outcome. A-type-selective or management-selective benefit of antihypertensive agents may exist in aortic dissection patients.

## Data Availability

The datasets presented in this article are not readily available because only citizens of the Republic of China who fulfill the requirements of conducting research projects are eligible to apply for the National Health Insurance Research Database (NHIRD). The use of the NHIRD is limited to research purposes only. Applicants must follow the Computer-Processed Personal Data Protection Law (http://www.winklerpartners.com/?p&equals;987) and related regulations of National Health Insurance Administration and NHRI. Requests to access the datasets should be directed to https://nhird.nhri.edu.tw/en.
