# Corrigendum: Prescription pattern and effectiveness of antihypertensive drugs in patients with aortic dissection who underwent surgery

**DOI:** 10.3389/fphar.2024.1438588

**Published:** 2024-06-18

**Authors:** Kuang-Ming Liao, Chuan-Wei Shen, Yun-Hui Huang, Chun-Hui Lu, Hsuan-Lin Lai, Chung-Yu Chen

**Affiliations:** ^1^ Chi Mei Medical Center, Chiali, Taiwan; ^2^ School of Pharmacy, Kaohsiung Medical University, Kaohsiung, Taiwan; ^3^ Department of Pharmacy, Taipei Tzu Chi Hospital, Buddhist Tzu Chi Medical Foundation, New Taipei City, Taiwan; ^4^ Institute of Medical Science and Technology, National Sun Yat-Sen University, Kaohsiung, Taiwan; ^5^ Division of Pharmacy, Kaohsiung Armed Forces General Hospital, Kaohsiung, Taiwan; ^6^ Department of Medical Research, Kaohsiung Medical University Hospital, Kaohsiung, Taiwan; ^7^ Department of Pharmacy, Kaohsiung Medical University Hospital, Kaohsiung, Taiwan

**Keywords:** aortic dissection, prescription patterns, antihypertensive drugs, *β*-blockers, angiotensinconverting enzyme inhibitor, angiotensin receptor blocker, calcium channel blocker

In the published article, an author name was incorrectly written as “Kung-Ming Laio”. The correct spelling is “Kuang-Ming Liao”.

The authors apologize for this error and state that this does not change the scientific conclusions of the article in any way. The original article has been updated.

