# Supplementary material for: Prescription pattern and effectiveness of antihypertensive drugs in patients with aortic dissection who underwent surgery
Source: Front Pharmacol. 2023 Nov 10;14:1291900. doi: 10.3389/fphar.2023.1291900 (PMC10667675; doi:10.3389/fphar.2023.1291900)
Supplement: Supplementary file 1 [file Table1.docx]

**Appendix 1. Description results of outpatient visit time and adjusted hazard ratios of outpatient visit time and classes for the composite outcome in operated aortic dissection patients.**

|  |  | Outpatient visit time | | | |  | Class | |
| --- | --- | --- | --- | --- | --- | --- | --- | --- |
|  | N | Mean (SD) | ***P*** value | Adjusted HR | ***P*** value |  | Adjusted HR | ***P*** value |
| Class 0 | 180 | 1.76 (2.50) | <0.001 | 0.89 (0.83-0.97) | 0.004 |  | 1.68 (1.14-2.48) | 0.009 |
| Class 1 | 180 | 4.16 (3.06) |  |  |  |  | ref |  |
| Class 2 | 574 | 3.66 (2.93) | 0.126 | 1.00 (0.96-1.04) | 0.953 |  | 1.07 (0.85-1.34) | 0.588 |
| Class 1 | 574 | 3.93 (3.05) |  |  |  |  | ref |  |
| Class 3 | 475 | 3.99 (2.97) | 0.407 | 1.01 (0.96-1.06) | 0.792 |  | 0.77 (0.58-1.01) | 0.057 |
| Class 1 | 475 | 3.83 (2.97) |  |  |  |  | ref |  |
| Class 4 | 218 | 3.91 (2.66) | 0.490 | 0.92 (0.84-1.01) | 0.081 |  | 1.07 (0.68-1.68) | 0.781 |
| Class 1 | 218 | 3.74 (2.46) |  |  |  |  | ref |  |
