# Supplementary material for: Prescription pattern and effectiveness of antihypertensive drugs in patients with aortic dissection who underwent surgery
Source: Front Pharmacol. 2023 Nov 10;14:1291900. doi: 10.3389/fphar.2023.1291900 (PMC10667675; doi:10.3389/fphar.2023.1291900)
Supplement: Supplementary file 2 [file Table2.docx]

**Appendix 2. Subgroup analysis of composite outcome of different combinations of antihypertensive drugs in operated type A aortic dissection patients**

|  | **N** | **Events** | **PY** | **Rate**  **(%) ^a^** | **Crude HR**  **(95% CI)** | ***P* value** | **Adjusted HR ^b^**  **(95% CI)** | ***P* value** |
| --- | --- | --- | --- | --- | --- | --- | --- | --- |
| **Class 1** |  |  |  |  |  |  |  |  |
| β-blocker | 253 | 53 | 1045 | 5.07 | 0.72 (0.41-1.27) | 0.256 | 1.17 (0.61-2.22) | 0.640 |
| CCB | 84 | 20 | 333 | 6.01 | 0.84 (0.43-1.62) | 0.601 | 1.34 (0.66-2.72) | 0.424 |
| RAS | 32 | 6 | 132 | 4.53 | 0.65 (0.26-1.67) | 0.373 | 0.81 (0.3-2.2) | 0.680 |
| Others | 61 | 16 | 226 | 7.08 | 1 (reference) |  | 1 (reference) |  |
| **Class 2** |  |  |  |  |  |  |  |  |
| β-blocker +CCB | 241 | 58 | 975 | 5.95 | 1.27 (0.51-3.18) | 0.605 | 1.3 (0.51-3.35) | 0.586 |
| β-blocker +Others | 128 | 35 | 508 | 6.89 | 1.51 (0.59-3.86) | 0.387 | 1.48 (0.57-3.85) | 0.423 |
| β-blocker +RAS | 114 | 29 | 419 | 6.92 | 1.5 (0.58-3.87) | 0.405 | 1.55 (0.58-4.14) | 0.383 |
| CCB+Others | 56 | 15 | 233 | 6.45 | 1.4 (0.51-3.85) | 0.515 | 1.26 (0.44-3.58) | 0.664 |
| CCB+RAS | 48 | 11 | 194 | 5.66 | 1.22 (0.42-3.5) | 0.717 | 1.11 (0.37-3.3) | 0.851 |
| RAS+Others | 22 | 5 | 110 | 4.53 | 1 (reference) |  | 1 (reference) |  |
| **Class 3** |  |  |  |  |  |  |  |  |
| β-blocker +CCB+Others | 126 | 23 | 526 | 4.37 | 0.86 (0.41-1.8) | 0.68 | 0.93 (0.42-2.04) | 0.847 |
| β-blocker +CCB+RAS | 245 | 36 | 1027 | 3.50 | 0.69 (0.34-1.39) | 0.299 | 0.83 (0.39-1.75) | 0.625 |
| β-blocker +RAS+Others | 86 | 21 | 329 | 6.38 | 1.25 (0.59-2.66) | 0.556 | 1.37 (0.62-3.03) | 0.434 |
| CCB+RAS+Others | 48 | 10 | 197 | 5.07 | 1 (reference) |  | 1 (reference) |  |
| ^a^ Rate was calculated as events divided by person-years, presented as %.  ^b^ Adjustment for covariates selected by stepwise multiple regression analyses and important risk factors associated with aortic dissection, including age, sex, comorbidities of hypertension, hyperlipidemia, diabetes mellitus, heart failure, coronary artery disease, cerebrovascular disease, chronic kidney disease, chronic obstructive pulmonary disease.  * *P*<0.016 in class 1 and class 3, *P*<0.01 in class 2 (adjusted the multiple comparisons with Bonferroni correction)  Abbreviations: HR, hazard ratio. PY, person-year. | | | | | | | | |
